# Lineage-specific variation in the evolutionary stability of coral photosymbiosis

**DOI:** 10.1126/sciadv.abh4243

**Published:** 2021-09-22

**Authors:** Jordan A. Gault, Bastian Bentlage, Danwei Huang, Alexander M. Kerr

**Affiliations:** 1 Marine Laboratory, University of Guam, 303 University Dr., Mangilao, GU 96913, USA.; 2 Department of Biological Sciences, Tropical Marine Science Institute, and Centre for Nature-based Climate Solutions, National University of Singapore, Singapore 117558, Singapore.

## Abstract

More than half of reef-building corals (Scleractinia) participate in a nutritional symbiosis, known as photosymbiosis, with photosynthetic dinoflagellates that ranges from obligate to facultative dependence. Fitting hidden-rates models allowing among-lineage variation in the rate of trait evolution to supertree and molecular phylogenies of Scleractinia, we reconstruct the history of photosymbiosis within Scleractinia and characterize its evolutionary stability. We find that most lineages of scleractinians are extraordinarily stable for the trait, evincing no instances of loss, but that in some clades photosymbiosis is more labile, thus providing a framework for comparative studies to further our mechanistic understanding of the factors that shape the evolutionary fates of scleractinian photosymbiosis.

## Teaser

The evolutionary stability of photosymbiosis differs among lineages of reef-building corals.

## INTRODUCTION

Symbioses are an important source of evolutionary novelty (*1*) that have played major roles in the history of life (*2*). However, the evolutionary patterns of symbioses and the evolutionary fates of their participants are not well understood (*3*). In scleractinian corals, photosymbiosis between the coral host and intracellular photosynthetic algae represents a nutritional mutualism that benefits both partners. Approximately half of the more than 1600 species of scleractinian corals participate in photosymbiosis wherein they host photoautotrophic dinoflagellate symbionts of the family Symbiodiniaceae (*4*, *5*), more commonly referred to as zooxanthellae. These photosymbiotic, or “zooxanthellate,” corals are limited to the photic zone where their symbionts provide photosynthate in exchange for nutrients (*6*) and support positive chemical feedback that accelerates calcification of coral skeletons (*7*), thus facilitating accretion of expansive reefs in shallow tropical and subtropical waters that shelter a quarter to a third of all marine species (*8*). More than 700 scleractinian corals are nonphotosymbiotic (“azooxanthellate”) and are more widely distributed latitudinally and bathymetrically (*9*). Twelve species are known to be facultatively symbiotic (“apozooxanthellate”), photosymbiotic in suitable habitats and nonphotosymbiotic otherwise (*10*, *11*). Despite the ecological significance of coral photosymbiosis, the stability and maintenance of this trait over evolutionary time scales remain obscure. Previous studies have reconstructed the evolutionary history of photosymbiosis, and there is evidence that the rate of evolution is not constant throughout the phylogeny of Scleractinia (*12*, *13*). However, information on which scleractinian lineages differ in the rate of evolution of photosymbiosis has been lacking. Moreover, because of the paucity of molecular sequence data for deep-water corals, previous studies included less than half of the extant coral diversity with a bias toward shallow-water, photosymbiotic species. To maximize taxon sampling and decrease bias toward photosymbiotic coral species, we evaluated the evolutionary stability of photosymbiosis using the posterior set of the most comprehensive supertree phylogeny of Scleractinia, which includes 1471 of the 1619 recognized species (*14*). In addition, we confirmed our results using the posterior set of a 579-species multilocus molecular phylogeny (*15*). Using “hidden-rates” models (HRMs) that accommodate lineage-specific variation in the rate of trait evolution (*16*), we reconstructed the evolutionary history of photosymbiosis in Scleractinia and identified lineages that appear evolutionarily stable for photosymbiosis, evincing no evidence of gain and loss of the trait, and those that appear unstable with the trait being gained or lost frequently. Using the HRM framework, we also calculated the probability that a given extant species is stable or unstable for the trait, thus providing a road map for comparative studies to further our mechanistic understanding of the factors that shape the evolutionary fates of coral photosymbiosis and symbioses in general.

## RESULTS

### Lineage-specific rates of gain and loss characterize the evolution of photosymbiosis

**Fig. 1.**
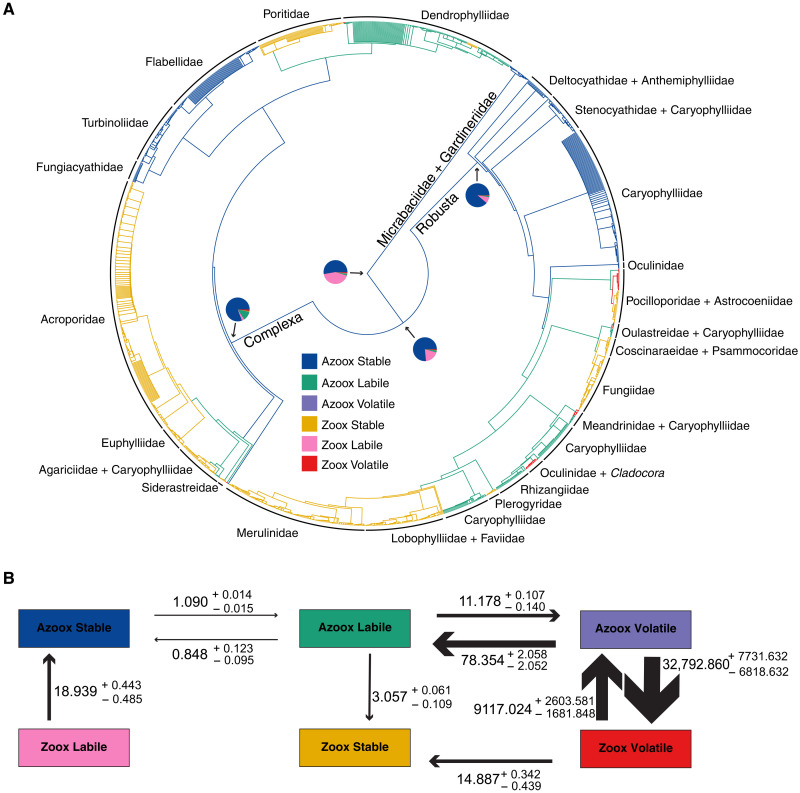
Supertree ancestral state reconstruction and transition rates estimated under the HRM + 3. (**A**) Ancestral state reconstruction across the 100 supertree phylogenies under the HRM + 3 summarized on the 95% consensus tree. Pie charts at selected nodes show the probability of being in each state/rate category. Branches are painted according to the most likely state at their ancestral node. (**B**) Schematic of the transition matrix of the HRM + 3. Transition rates shown are the median rates estimated for the 100 supertree phylogenies. Errors shown are the bootstrapped 95% quantiles. Values are multiplied by 1000 to aid interpretation. The width of the arrows corresponds to the relative magnitude of the rates.

HRMs allow for lineage-specific variation in the rate of evolution by partitioning the model into multiple rate categories that can be fit to different parts of the phylogeny. For example, in an HRM with two rate categories (HRM + 2), there will be a “slow” rate category in which transition rates are relatively low and a “fast” rate category in which transition rates are relatively high. Fitting HRMs with one to four rate categories across the supertree phylogenies, we found that the model that best explains the evolution of photosymbiosis is an HRM with three rate categories (HRM + 3; Akaike Information Criterion corrected (AICc) weight of 98.27%; Table 1). Under the HRM + 3, there are three distinct categories of evolutionary stability of photosymbiosis: a “Stable” category, where transitions between photosymbiotic states never occur; a “Labile” category, where transitions between photosymbiotic states are more likely; and an extremely labile, or “Volatile,” category, where transitions between photosymbiotic states are very likely (Fig. 1B). Note that in this context, stability is a phylogenetic concept and refers to the probability of a lineage retaining a trait over evolutionary time.

**Table 1.** Summary of AICc scores for each model fit to the 1000 supertree and 3361 molecular phylogenies. TH, time homogeneous.

**Model**	**Rate categories**	**Parameters**	**Supertree phylogeny**		**Molecular phylogeny**	
			**Mean AICc**	**AICc weight (%)**	**Mean AICc**	**AICc weight (%)**
TH	1	2	335.560	0	181.522	0.213
HRM + 2	2	8	286.566	1.062	169.421	91.284
HRM + 3	3	14	277.511	98.268	173.923	9.503
HRM + 4	4	20	287.485	0.671	–	–

**Fig. 2.**
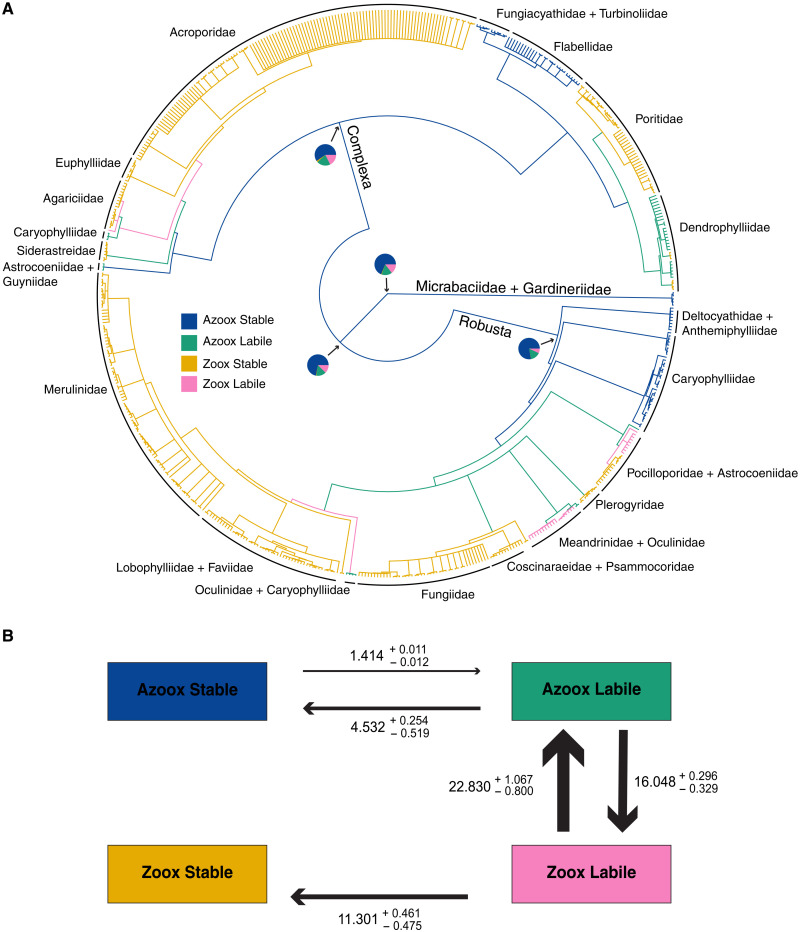
Molecular tree ancestral state reconstruction and transition rates estimated under the HRM + 2. (**A**) Ancestral state reconstruction of each state/rate category combination inferred across the 3361 molecular phylogenies under the HRM + 2. Phylogeny shown is the 95% consensus tree of all 3361 molecular phylogenies used for the analysis. The corallimorpharian outgroup is excluded from the plot for presentation. To calculate the probability at each internal node, the mean of each state/rate category across all phylogenies was calculated for all nodes that are bifurcating in the 95% consensus tree. Pie charts at selected nodes show the probability of being in each state/rate category. Branches are painted according to the state that is most likely at each internal node. (**B**) Schematic version of the transition matrix of the HRM + 2 fit to the 3361 molecular phylogenies. Transition rates printed here are the median of the transition rates estimated for the 3361 molecular phylogenies. Errors printed here represent the 95% quantile around the median as estimated via bootstrapping. Median values and errors are multiplied by 1000 to aid interpretation. The width of the arrows corresponds to the relative magnitude of the rates.

We found that the model that best explains the evolution of photosymbiosis across the molecular phylogenies is an HRM with two rate classes (HRM + 2; AICc weight of 90.28%; Table 1). Because the HRM + 2 was found to be a better fit than the HRM + 3, we did not fit an HRM with four rate categories. Under the HRM + 2, there are two distinct categories of evolutionary stability of photosymbiosis: a Stable category where transitions between photosymbiotic states never occur and a Labile category where transitions between photosymbiotic states are more likely (Fig. 2B). Although the best fit model for the molecular tree has only two rate categories, it is qualitatively very similar to the HRM + 3 model that fit the supertree best. In both models, photosymbiosis is never gained directly from the stable azooxanthellate state (Azoox Stable; Figs. 1B and 2B). Rather, Azoox Stable lineages must first transition to the Labile rate category. The Labile rate category is characterized by high rates of transition between photosymbiotic states (Fig. 2B). Under the HRM + 2, Azoox Stable lineages can transition to the unstable azooxanthellate state (Azoox Labile), after which unstable photosymbiosis can be gained (Zoox Labile; Fig. 2B). From the Zoox Labile state, lineages can then acquire stable photosymbiosis (Zoox Stable), after which photosymbiosis is not lost (Fig. 2B).

Compared to the HRM + 2, the HRM + 3 contains an additional Volatile rate category that is characterized by extremely high rates of transition between photosymbiotic states and high rates of transition away from the rate category (Fig. 1B). Unlike the HRM + 2, the Zoox Stable state can be gained directly from the Azoox Labile state (Fig. 1B). Alternatively, lineages can transition from Azoox Labile to the volatile state where transitions between nonphotosymbiotic (Azoox Volatile) and photosymbiotic (Zoox Volatile) states can occur rapidly (Fig. 1B). Zoox Volatile lineages can transition to the Zoox Stable state where, as in the HRM + 2, photosymbiosis is not lost (Fig. 1B). In the HRM + 3, the placement of the Zoox Labile state within the model and position on the phylogeny are subject to phylogenetic uncertainty (figs. S1 to S3; see Supplementary Text and the “Ancestral state reconstruction” section below).

### Azooxanthellate lineages differ in the rate of gain of photosymbiosis

Using rates estimated from the HRMs fit to their respective phylogenies, we estimated the ancestral states at interior nodes of the phylogenies to identify lineages with different rates of gain and loss of photosymbiosis. For each species, we also calculated the probability of occupying a particular rate class for the observed trait. Reconstructing the ancestral states at interior nodes across the posterior sets of the supertree (Fig. 1A and fig. S6) and molecular (Fig. 2A and fig. S7) phylogenies, we found that Complexa, Robusta, Micrabaciidae + Gardineriidae, and the Complexa/Robusta split were confidently reconstructed as ancestrally Azoox Stable, with a probability of being in the Azoox Stable state greater than 75% at each node [Pr(Azoox Stable) > 0.75]. Across the posterior set of molecular phylogenies, the ancestral state of Scleractinia was confidently reconstructed as Azoox Stable [Pr(Azoox Stable) > 0.75; Fig. 2A]. However, the ancestral state of Scleractinia is uncertain across the supertree phylogenies [Pr(Azoox Stable) = 0.53; Fig. 1A]. The next most likely state at the root is the Zoox Labile state [Pr(Zoox Labile) = 0.42]. The Zoox Labile state was not confidently reconstructed at any interior node of the tree [Pr(Zoox Labile) < 0.20 at all nodes other than the root], and no extant species were confidently inferred to be in the Zoox Labile state [Pr(Zoox Labile) < 0.2 for all species; table S1]. This is most likely because within the HRM + 3, the placement of the Zoox Labile state is subject to phylogenetic uncertainty: Across 158 of the 1000 supertree phylogenies, Azoox Labile is a transitional state toward the evolution of stable photosymbiosis and was not reconstructed at the root (figs. S1 and S2; see Supplementary Text). Of the 700 azooxanthellate species in the phylogeny, 399 were estimated to be in the Stable rate category [Pr(Azoox Stable) > 0.75; table S1]. Of the 125 azooxanthellate species in the molecular phylogeny, 86 were estimated to be in the Stable rate category [Pr(Stable) > 0.75; table S2]. In both phylogenies, Azoox Stable species are found mostly in clades composed of Deltocyathidae, Caryophylliidae, Fungiacyathidae, Turbinoliidae, and Flabellidae, all of which retain the ancestral azooxanthellate trait.

On both the supertree and molecular phylogenies, transitions from Azoox Stable to Azoox Labile precede the evolution of photosymbiosis. Specifically, there were three such transitions, one within Robusta and two within Complexa (Figs. 1A and 2A). Of the 700 azooxanthellate species in the supertree phylogenies, 291 were estimated to be in the Labile rate category [Pr(Azoox Labile) > 0.75; table S1] and are found mostly in clades comprising the families Caryophylliidae, Rhizangiidae, and Dendrophylliidae. Many of these Azoox Labile species within Complexa (Caryophylliidae and Rhizangiidae) are not included in the molecular tree, and correspondingly fewer species were estimated to be in the Azoox Labile state. Of the 125 azooxanthellate species included in the molecular tree, only 12 (mostly caryophyliids) were estimated to be in the Labile rate category [Pr(Azoox Labile) > 0.75; table S2].

### Stable photosymbiosis has arisen multiple times

The Zoox Stable state has multiple, independent origins in the supertree and molecular phylogenies and once gained is never lost (Table 2). Within Robusta, four large clades independently gained the Zoox Stable state (Figs. 1A and 2A). The first is composed entirely of Pocilloporidae. The second consists of Coscinaraeidae, Fungiidae, and Psammocoridae. The third is composed of Plerogyridae. The fourth includes Lobophylliidae, Faviidae, and Merulinidae. Within Complexa, five clades independently gained the Zoox Stable state (Figs. 1A and 2A). The first is composed of Siderastreidae. The second is composed of Agariciidae. The third is composed of Euphylliidae and Acroporidae, although these could potentially represent two independent gains in the molecular phylogeny (fig. S7). The fourth gain is Poritidae, and the fifth is the genus *Turbinaria* within the Dendrophylliidae (although *Turbinaria* is not monophyletic in the molecular phylogenies, leading to two independent acquisitions). Of the 767 zooxanthellate species in the supertree phylogeny, 721 were estimated to be in the Stable rate category [Pr(Zoox Stable) > 0.75; table S1]. Of the 450 zooxanthellate species in the molecular phylogeny, 416 were estimated to be in the Stable rate category [Pr(Zoox Stable) > 0.75; table S2].

**Table 2.** Number of gains and losses of each state/rate category estimated across the 1000 supertree and 3361 molecular phylogenies. Ranges represent the 95% quantiles around median calculated using bootstrapping.

**State/rate** **category**	**Supertree phylogeny**		**Molecular phylogeny**	
	**Gains**	**Losses**	**Gains**	**Losses**
Azoox Stable	2	3	2–3	5
Zoox Stable	10	0	15	0
Azoox Labile	6	24	9	18
Zoox Labile	0	2	9–10	15–16
Azoox Volatile	10	0	–	–
Zoox Volatile	15–16	13–14	–	–

### Some lineages differ in the evolutionary stability of photosymbiosis

Several small clades were found to have high rates of gain and loss of photosymbiosis. Across the supertree phylogenies, five small clades within Robusta were reconstructed as Zoox Volatile (Fig. 1A): Astrocoeniidae, *Madracis* + *Palauastrea* (sister lineages to the Zoox Stable members of the Pocilloporidae), *Oulastrea* + *Heterocyathus*, Meandrinidae, and Oculinidae. Across the molecular phylogenies, the same clades were reconstructed as Zoox Labile with the exception of the genera *Oulastrea* and *Heterocyathus*. Across the supertree phylogenies, nine scattered species are inferred to be Zoox Volatile in Complexa (table S1): *Stephanocoenia intersepta* (Astrocoeniidae), *Helioseris cucullata* (Agariciidae), and five species within Dendrophylliidae—*Balanophyllia* (*Balanophyllia*) *europaea*, *Duncanopsammia axifuga*, *Dichopsammia granulosa*, *Heteropsammia eupsammides*, and *Heteropsammia cochlea*. Across the molecular phylogenies, two species were inferred to be Zoox Labile in Complexa (table S2): *H. cucullata* (Agariciidae) and *H. cochlea* (Dendrophylliidae). Of the 767 zooxanthellate species in the supertree phylogeny, 33 were estimated to be in the Volatile rate category [Pr(Zoox Volatile) > 0.75; table S1]. Of the 450 zooxanthellate species in the molecular phylogeny, 16 were estimated to be in the Labile rate category [Pr(Zoox Labile) > 0.75; table S2]. Notably, in both supertree and molecular phylogenies, nearly all facultative species were estimated to be in unstable rate categories (tables S1 and S2). Across the molecular phylogenies, all facultative species were estimated to be in the Labile rate category [Pr(Zoox Labile) > 0.75; table S2]. Analogous to these results, across the supertree phylogenies, all facultative species were estimated to be in the Volatile rate category [Pr(Zoox Volatile) > 0.75] with the exception of *D. granulosa* [Pr(Zoox Volatile) = 0.66; table S2]. To verify that facultative corals were not driving the reconstruction of Volatile clades in the supertree and Labile clades in the molecular phylogeny, we repeated the analyses with facultative corals pruned from 100 randomly subsampled phylogenies and obtained the same results (figs. S4 and S5). Across the supertree phylogenies, the Azoox Volatile state was not reconstructed confidently at any internal nodes [Pr(Azoox Volatile) < 0.25 for all nodes; table S1]. Only 10 species were estimated to be in the Azoox Volatile state [Pr(Azoox Volatile) > 0.75; table S1]. All 10 species represent losses of photosymbiosis and were found in clades reconstructed as Zoox Volatile.

## DISCUSSION

### Ancestral state of Scleractinia

Fitting HRMs to both supertree and molecular phylogenies, we found that the rate of evolution of photosymbiosis varies among lineages. The best fit HRMs contain multiple rate categories to account for variability in the evolutionary stability of photosymbiosis, illuminating differences in the probability of a lineage to gain, retain, or lose this trait. Using rates estimated under the HRMs, we reconstructed the ancestral states at interior nodes across the posterior sets of the supertree (Fig. 1A and fig. S6) and molecular (Fig. 2A and fig. S7) phylogenies and identified lineages that differ in the rate of gain and loss of photosymbiosis. Last, for each species, we calculated the probability of occupying a particular rate category, thus identifying species that are likely to be stable or unstable for their observed trait (tables S1 and S2). Across both phylogenies, we found that Complexa, Robusta, Micrabaciidae + Gardineriidae, and the Complexa/Robusta split are confidently reconstructed as ancestrally Azoox Stable [Pr(Azoox Stable) > 0.75; table S1] and that several large clades retain this ancestral state (Figs. 1A and 2A). Across the posterior set of molecular phylogenies, the ancestral state of Scleractinia is confidently reconstructed as Azoox Stable [Pr(Azoox Stable) > 0.75; Fig. 2A and fig. S7]. In contrast, the ancestral state of Scleractinia is uncertain across the supertree phylogenies, with Azoox Stable [Pr(Azoox Stable) = 0.53; Fig. 1A and fig. S6] and Zoox Labile [Pr(Zoox Labile) = 0.42] being nearly equivocal states. Recent ancestral state reconstructions indeed inferred Scleractinia as ancestrally photosymbiotic (*17*, *18*), but both studies had relatively low taxon sampling within Scleractinia. Rather than suggesting a zooxanthellate origin for Scleractinia, the placement of Zoox Labile at the root may reflect an inability of the HRM + 3 to adequately fit the Labile category to any zooxanthellate clades. A number of our results suggest the latter. First, the ancestral state of Scleractinia was reconstructed as Azoox Stable across the molecular phylogenies that include a zooxanthellate corallimorpharian outgroup (omitted from figures for presentation). Second, the placement of the Azoox Labile state and its role in the model are subject to phylogenetic uncertainty. Across 158 of the 1000 supertree phylogenies, Azoox Labile is a transitional state toward the evolution of stable photosymbiosis and was not reconstructed at the root (figs. S1 and S2). Last, similar uncertainty was introduced at the root of the molecular phylogenies if ancestral states were reconstructed under the HRM + 3 (fig. S3). Together, this suggests that under the HRM + 3, no clades fit the Zoox Labile category, which was thus placed at the deepest node of the phylogeny where it is rapidly lost and never regained (Fig. 1, A and B).

If the uncertainty at the root of the supertree phylogenies is considered an artifact of model complexity, then the inference of an azooxanthellate origin for the order based on the molecular phylogenies is in line with both phylogenetic and fossil evidence. The finding that the exclusively deep-water, azooxanthellate Micrabaciidae and Gardineriidae diverge before the Complex/Robust split and that many deep-water, azooxanthellate species diverge deeply within Complexa and Robusta supports the deep-water origin of Scleractinia (*19*, *20*). Moreover, molecular clock estimates date the origin of the order deep within the Paleozoic, well before the appearance of the well-differentiated and diverse Triassic scleractinian fauna. This led to the suggestion that the Paleozoic scleractiniamorphs (*21*–*24*) are true scleractinians (*19*). These early scleractinians were solitary and most likely azooxanthellate deep-water inhabitants, supporting the argument for a nonphotosymbiotic origin of the order. Moreover, the first Triassic scleractinians were solitary and phaceloid, which suggests that they were azooxanthellate (*25*). More recently, Campoy *et al.* (*13*) reconstructed the ancestral state of the order as azooxanthellate. Given the findings of these previous studies and our results, the most recent common ancestor of living Scleractinia is likely azooxanthellate with photosymbiosis in extant scleractinian corals being a derived state.

### Key transitions toward the evolution of scleractinian photosymbiosis

Azoox Stable lineages show no propensity to directly evolve photosymbiosis and must first transition to a different rate category (Azoox Labile) before acquiring photosymbiosis (Figs. 1, A and B, and 2, A and B). Within the HRM framework, these rate categories are treated as unobserved states, which, biologically, are interpreted as correlating with some unobserved trait that affects the rate of evolution of the observed trait. In other words, the transition from Azoox Stable to Azoox Labile corresponds with the acquisition of some unknown, necessary trait that precedes the evolution of photosymbiosis. The evolution of coloniality has been proposed as a possible precursor to the evolution of photosymbiosis in scleractinian corals (*12*, *13*). Could the transition from Azoox Stable to Azoox Labile correspond to the evolution of coloniality? Campoy *et al.* (*13*) found that coloniality is a labile trait in azooxanthellate lineages, with relatively high rates of gain and loss. If the transition from Azoox Stable to Azoox Labile corresponds with the acquisition of coloniality, then we would expect to see a high number of transitions between these states throughout the phylogeny. However, we observed only three transitions from Azoox Stable to Azoox Labile and, at most, two transitions from Azoox Labile to Azoox Stable. It is therefore unlikely that the transition to Azoox Labile corresponds to the evolution of coloniality.

We suggest that the transitions to Azoox Labile indicate the time points in the evolutionary history of corals during which the molecular and cellular mechanisms evolved that allow mediation of the coral host-photosymbiont relationship. The processes mediating coral photosymbiosis overlap with innate immunity pathways (*26*–*31*), which are involved in mediating interactions with both harmful and beneficial microbes. In scleractinian corals, photosymbiosis may have been facilitated by the diversification of these innate immunity pathways, and the transition to Azoox Labile may be driven by extensive gene duplications, followed by the exaptation of immune pathways involved in maintaining microbiome communities (*27*).

### Evolutionary stability of scleractinian photosymbiosis

Most photosymbiotic corals are found in clades that are stable for the trait (Zoox Stable). Stable photosymbiosis has evolved independently multiple times within both Complexa and Robusta. Almost all families in Zoox Stable clades appear in the fossil record by the late Cretaceous (*25*, *32*, *33*), while some (including Merulinidae, Lobophylliidae, and Faviidae) could potentially be Jurassic in origin, coinciding with the origin and diversification of Symbiodiniaceae (*4*). This indicates that photosymbiosis has been remarkably stable in most clades of corals over evolutionary time scales. Some smaller clades, however, show higher rates of gain and loss of photosymbiosis (e.g., parts of Pocilloporidae in Robusta and Dendrophylliidae in Complexa). In these clades, extant photosymbiotic species are inferred to be in the Volatile photosymbiotic rate category in the supertree and the corresponding Labile category in the molecular tree (tables S1 and S2). High rates of gains and losses of photosymbiosis within these clades suggest that photosymbiotic species in the Labile and Volatile rate categories are not obligately dependent on their symbionts in the same way that Zoox Stable corals are, allowing for loss and subsequent regain of photosymbiosis over evolutionary time scales. This is supported by the fact that all facultative species are estimated to be in the Labile or Volatile rate category.

The question that begs answering is why photosymbiosis remains stable in most scleractinian clades but appears labile in others. Nutritional symbioses, such as photosymbiosis, are thought to be prone to abandonment if one partner can acquire sufficient nutrition from the environment (*3*). Moreover, conflicts of interest between symbiotic partners are thought to make symbioses unstable over evolutionary time scales (*34*). Vertical transmission and intracellular integration of symbionts are important means to stabilize the relationship between host and symbiont (*34*). In contrast, most scleractinian corals horizontally acquire their photosymbionts from the environment (*35*) and host multiple symbiont strains (*36*, *37*), both factors thought to destabilize symbiotic interactions (*34*). It is therefore unexpected that no losses of photosymbiosis are observed in most photosymbiotic lineages. Understanding the relationship between transmission mode and evolutionary stability of scleractinian photosymbiosis will require consideration of the issue from the perspective of both the host and the symbionts. For instance, vertically transmitted photosymbionts have higher thermal tolerance and higher host specificity, which may result in symbioses that are more resistant to climate change (*38*). However, vertical transmission comes with concomitant costs for the coral host including increased risk of thermal and ultraviolet damage to broadcast larvae, which may favor horizontal transmission among broadcast spawners (*35*). Investigations of the network of interactions between scleractinian hosts and their symbionts have shown that both horizontal and vertical transmitters host communities of symbionts that range from low to high host specificity (*37*). The presence of symbionts with high host specificity could lead to multigenerational host-symbiont fidelity that fosters mutual dependence regardless of transmission mode. However, what is the nature of this dependence?

It is plausible that gene losses and associated losses in the metabolic capacity of the host play an important role in the development of obligate photosymbiosis in scleractinian corals, similar to other symbiotic systems (*2*). For example, corals within Robusta appear to have retained an ancestral histidine biosynthesis pathway, while corals within Complexa lost this pathway independently from bilaterian animals (*39*). Corals within Complexa also appear unable to biosynthesize cysteine, indicating at least some degree of metabolic dependence on their photosymbionts (*40*). Detailed examination of the metabolic complementarity between coral hosts and their respective photosymbiont strains [e.g., (*40*)] will be necessary to elucidate the nature of the photosymbiotic partnership across coral lineages. It will be important to investigate the genomic architecture underlying host-symbiont interactions in lineages with different rates of gain and loss of photosymbiosis. At this point, we lack broad taxonomic sampling of sequenced genomes for host-symbiont pairs necessary for further inquiry (*41*). The phylogenetic framework presented here allows identification of the next coral models from lineages differing in the rates of gain and loss of photosymbiosis to determine the proximate cause governing the evolutionary stability of photosymbiosis in corals.

### The role of extinction in understanding scleractinian photosymbiosis

The methods used here for rate estimation and ancestral state reconstruction assume minimal extinction or equal extinction risk across all states. Differential extinction of photosymbiotic versus nonphotosymbiotic corals could bias ancestral state reconstructions, especially at deeper nodes. There is evidence that photosymbiosis evolved in the Triassic (*42*, *43*). Scleractinians suffered a major bottleneck at the end of the Triassic, during which nearly all species went extinct, including all photosymbiotic lineages (*44*, *45*). The placement of these early photosymbiotic lineages in the phylogeny could potentially alter the reconstructions at deeper nodes in the tree. Because of homoplasy in morphological characters, integrating fossil specimens into the phylogenetic framework of Scleractinia remains challenging (*46*, *47*). The inclusion of fossil specimens in future phylogenies, if possible, will be an important step in reducing extinction as a source of potential bias. Furthermore, investigations that combine fossil and phylogenetic evidence to elucidate the timing and geographical locations of the appearance of photosymbiotic clades will be valuable. For example, corals in Robusta fared better than corals within Complexa after the end-Triassic extinction, which could have been related to their ability to seek refuge in deep water or high latitudes within the newly forming Atlantic ocean (*48*). The higher number of Zoox Volatile species found in Robusta versus Complexa could reflect an evolutionary history of adaptations to high latitude and low irradiance during periods of environmental change.

## MATERIALS AND METHODS

### Phylogenetic trees

We used two sets of phylogenetic trees for our analyses. First, we used a published posterior set of 1000 time-calibrated, fully resolved supertree phylogenies of 1547 species (*14*). This is the most complete phylogeny for Scleractinia (96% of extant species) and is composed of a backbone molecular phylogeny of 474 species (based on seven mitochondrial DNA markers), 13 morphological trees, and a taxonomic tree. The posterior supertree set is composed of 755 zooxanthellate and 700 azooxanthellate species. See (*14*, *49*, *50*) for a detailed description of the methods used to construct the supertree phylogeny.

Because the molecular source tree for the supertree uses only linked mitochondrial markers, we also used a 579-species molecular phylogeny constructed using both partial mitochondrial DNA [12*S* rDNA (ribosomal DNA), 16*S* rDNA, adenosine 5′-triphosphate synthase subunit 6, cytochrome c oxidase subunit 1, control region, cytochrome b, and NADH (reduced form of nicotinamide adenine dinucleotide) dehydrogenase subunit 5] and partial nuclear DNA (18*S* rDNA, 28*S* rDNA, histone H3, internal transcribed spacers, and Pax-C 46/47 intron) markers (*15*). This set is composed of 442 zooxanthellate species but only 125 azooxanthellate species, although all three basal-most clades—Complexa, Robusta, and Micrabaciidae + Gardineriidae [= “basal clade” sensu (*19*)]—are recovered. Eight Markov chain Monte Carlo runs were conducted in BEAST2 (*51*). Each chain ran for 50 million iterations and sampled every 1000 iterations to ensure lack of autocorrelation among trees. The first 10% of each chain was discarded as burn-in, and the rest were combined for a posterior distribution of 360,000 trees. This set was further thinned to an effective sample size of 3361 trees using the R (*52*) package coda (*53*).

We then standardized nomenclature across trees using the taxonomy from the World Register of Marine Species (WoRMS) (*54*) accessed via the R package “worrms” (*55*). After pruning synonyms, nomina nuda, and nomina dubia, the 1472 species remained in the supertree posterior set. However, no species needed to be pruned from the molecular posterior set.

### Trait data

Each species is scored as being azooxanthellate, obligately zooxanthellate, or facultatively zooxanthellate (tables S3 and S4) based on data obtained from the Coral Trait Database (*5*), WoRMS, Cairns’ online appendix of azooxanthellate corals (*9*), or the original species description. Five species [*Astreopora acroporina*, *Astreopora cenderawasih*, *Astreopora monteporina*, *Meandrina jacksoni*, and *Stephanocyathus* (*Stephanocyathus*) *isabellae*] lacked data on symbiotic state and were pruned from the phylogenies.

### HRMs and rate estimation

We used the HRMs of Beaulieu *et al.* (*16*) to test whether the rate of evolution of photosymbiosis varies throughout the tree. HRMs allow for lineage-specific variation in the rate of evolution by partitioning the model into multiple rate categories that can be fit to different parts of the phylogeny. These rate categories are treated as unobserved, or “hidden,” states. For an HRM with multiple rate categories, a species in an observed state (such as zooxanthellate) has uniform prior probability of being in each of the unobserved rate categories. Within each rate category, there can be unequal transition rates between the binary character states. Transitions can occur between states within a rate category or between rate categories within a given state. Because rates are instantaneous, simultaneous transitions between state and rate categories cannot occur. Biologically, the rate categories can be thought of as correlating with some unobserved trait that affects the rate of evolution of the observed trait. The rate categories are not specified a priori but are estimated from the data, allowing one to identify areas of the tree where evolution proceeds at different rates.

We used the R package corHMM (*56*) to fit HRMs with one to four rate categories (table S1) to each of the 1000 supertree phylogenies and HRMs with one to three rate categories to the 3361 molecular phylogenies via maximum likelihood. To broadly sample parameter space, the transition rates for each model were estimated over 100 random restarts. HRM fit was computed using the computational resources provided by the Open Science Grid (*57*, *58*). We then calculated the median value of the estimated transition rates over the subsampled phylogenies. The 95% confidence intervals were calculated by bootstrapping the median 100,000 times. We used the mean AICc for small sample size to assess each model’s fit across the subsampled phylogenies.

### Ancestral state reconstruction

To reconstruct the evolutionary history of photosymbiosis and identify areas of the phylogeny with differing rates of evolution, we calculated the marginal probabilities of each state at internal nodes of the supertree and molecular phylogenies using the corHMM package. We also calculated the probability of each extant species being in a particular rate category for its observed trait. We used a flat prior on the root where each state/rate category combination is equally likely. To incorporate phylogenetic uncertainty, we performed ancestral-state reconstruction for each of the posterior trees, with results summarized on a 95% consensus tree. For each bifurcating node in the consensus tree, we calculated the mean likelihood of being in each state/rate category across the corresponding nodes in the posterior set. Also, for each extant species, we calculated the mean likelihood of being in each rate category across the posterior set.

To estimate the minimum number of transitions between states and rate categories, we used the marginal probabilities at the internal nodes and tips of the phylogeny. We assigned to each tip and internal node the state and rate category with the highest probability. Then, assuming that only one change can occur per branch, we summed the gains and losses of each state and rate category. This gives the minimum number of transitions over the phylogeny implied by the marginal ancestral state reconstruction. To incorporate phylogenetic uncertainty, we calculated the median number of changes across the posterior set. We calculated 95% confidence intervals by bootstrapping the median 100,000 times.

### Sensitivity to state assignment

All facultative corals were found in clades inferred to be in the Volatile or Labile rate categories. Hence, to determine whether facultative corals were driving the rate assignment of these clades, as well as overall model structure, we pruned all facultative species from the phylogenies and reran the corHMM analyses for the first three models [TH (time homogeneous), HRM + 2, and HRM + 3] using 100 trees subsampled from both the supertree and molecular phylogenies. We then performed ancestral state reconstruction under the best-fit model for supertree and molecular phylogenies. Ancestral state reconstructions as summarized across the posterior set samples of the supertree and molecular phylogenies are shown in figs. S4 and S5, respectively. The removal of facultative corals from the phylogenies did not substantially alter the results or their interpretation.

## Supplementary Materials

### This PDF file includes:

Supplementary Text Figs. S1 to S5 Legends for figs. S6 and S7 Legends for tables S1 and S2 References
