# Supplementary material for: Lineage-specific variation in the evolutionary stability of coral photosymbiosis
Source: Sci Adv. 2021 Sep 22;7(39):eabh4243. doi: 10.1126/sciadv.abh4243 (PMC8457658; doi:10.1126/sciadv.abh4243)
Supplement: Supplementary file 1 — Supplementary Text Figs. S1 to S5 Legends for figs. S6 and S7 Legends for tables S1 and S2 References [file sciadv.abh4243_sm.pdf]

Science Advances
AAAS

Supplementary Materials for

Lineage-specific variation in the evolutionary stability of coral photosymbiosis

Jordan A. Gault*, Bastian Bentlage, Danwei Huang, Alexander M. Kerr

*Corresponding author. Email: jordan.gault@uni-oldenburg.de

Published 22 September 2021, *Sci. Adv.* **7**, eabh4243 (2021)
DOI: 10.1126/sciadv.abh4243

This PDF file includes:

Supplementary Text
Figs. S1 to S5
Legends for figs. S6 and S7
Legends for tables S1 and S2
References

Other Supplementary Material for this manuscript includes the following:

Figs. S6 and S7
Tables S1 and S2

Supplementary Text

Phylogenetic uncertainty

Phylogenetic uncertainty does lead to differences in the results across the 1000 supertree phylogenies and these results fall into two distinct categories (Figs. S2 and S3). The main difference between the two results is the relative placement and role of the Zoox Labile category within the model. This uncertainty in the placement of the Zoox Labile state appears to be driven by the inability of the model to fit the Zoox Labile state to any particular clade.

Across 842 phylogenies, Zoox Labile can transition directly into Azoox Stable after which Zoox Labile is never regained (Fig. S2). The ancestral state of the order is uncertain across these 842 phylogenies and the likelihood is split roughly equally between Azoox Stable and Zoox Labile. Figure S2 shows the ancestral state reconstructions for a sample of these 842 phylogenies. In most of the them, only the root is reconstructed as Zoox Labile (such as the leftmost two trees in Fig. S2). However, in others, the Agariciidae is reconstructed as Zoox Labile (such as the rightmost two trees in Figure S2).

Across 158 of the 1000 phylogenies, Zoox Labile is a transitional state in the evolution of stable photosymbiosis (Fig. S3). Transitions can occur between Zoox Labile and Azoox Labile. Zoox Labile is equally likely to transition into Azoox Labile and Zoox Stable, but if a transition to Zoox Stable occurs, the trait is never lost. Figure S3 shows the ancestral state reconstructions for a sample of these 158 phylogenies. Zoox Labile plays a more obvious transitional role in these reconstructions as its evolution generally precedes the evolution of Zoox Stable.

This uncertainty in the role and placement of the Zoox Labile state leads to the uncertainty in the ancestral state reconstruction of the root across the supertree phylogenies. Notably, across some of the individual ancestral state reconstructions on the posterior supertrees, Agariciidae is placed in the Zoox Labile category and is inferred to represent a loss after a shared gain with Acroporidae + Euphylliidae. There is in fact an azooxanthellate member of Agariciidae (*59*) which was not included in the phylogeny for this analysis. Its inclusion might place Agariciidae in the Zoox Labile category.

Similar uncertainty is introduced at the root of the molecular tree when fitting the HRM+3 to 100 randomly sampled molecular phylogenies. However, the root of the molecular tree is the ancestor of Scleractinia + Corallimorpharia so that Scleractinia is now confidently reconstructed as confidently Azoox Stable (Fig. S4). This behavior where Zoox Labile is placed at the deepest node in both the supertree and molecular phylogenies regardless of the node's identity suggests that the reconstruction of Zoox Labile at the root of the phylogenies is an artefact of model complexity rather than positive evidence of Zoox Labile at the root.

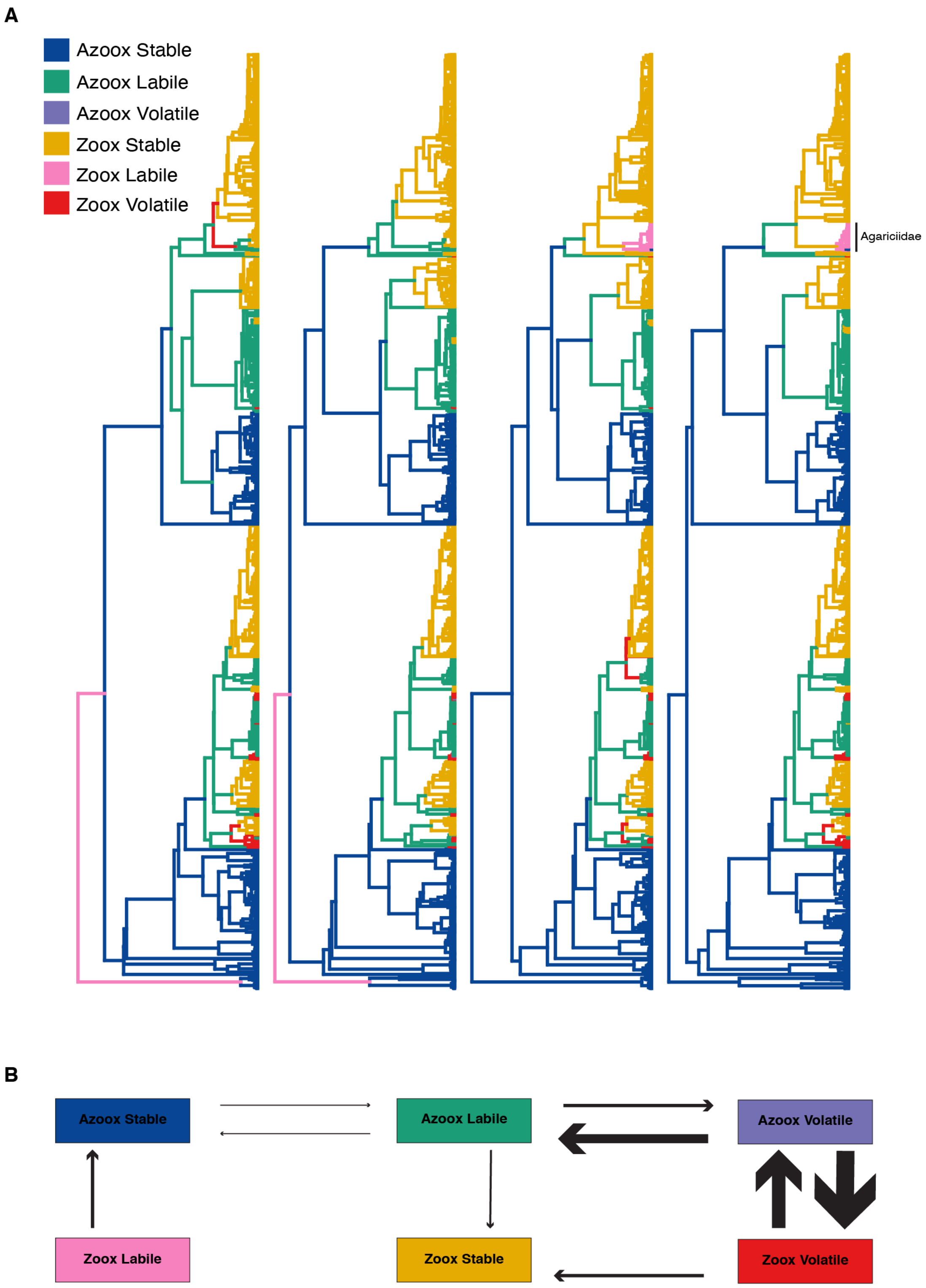

Fig. S1. Sample of the 842 supertree phylogenies where Azoox Labile is not a transition state under the HRM+3. (A) Individual ancestral state reconstructions of state/rate-category on

a subsample of the 842 supertree phylogenies for which Zoox Labile is not a transitional state. Branches are colored according to the most likely state at their ancestral node. **(B)** Corresponding rate diagram calculated from the sample. Rates have been omitted. Arrow width corresponds to the relative magnitudes of the rates.

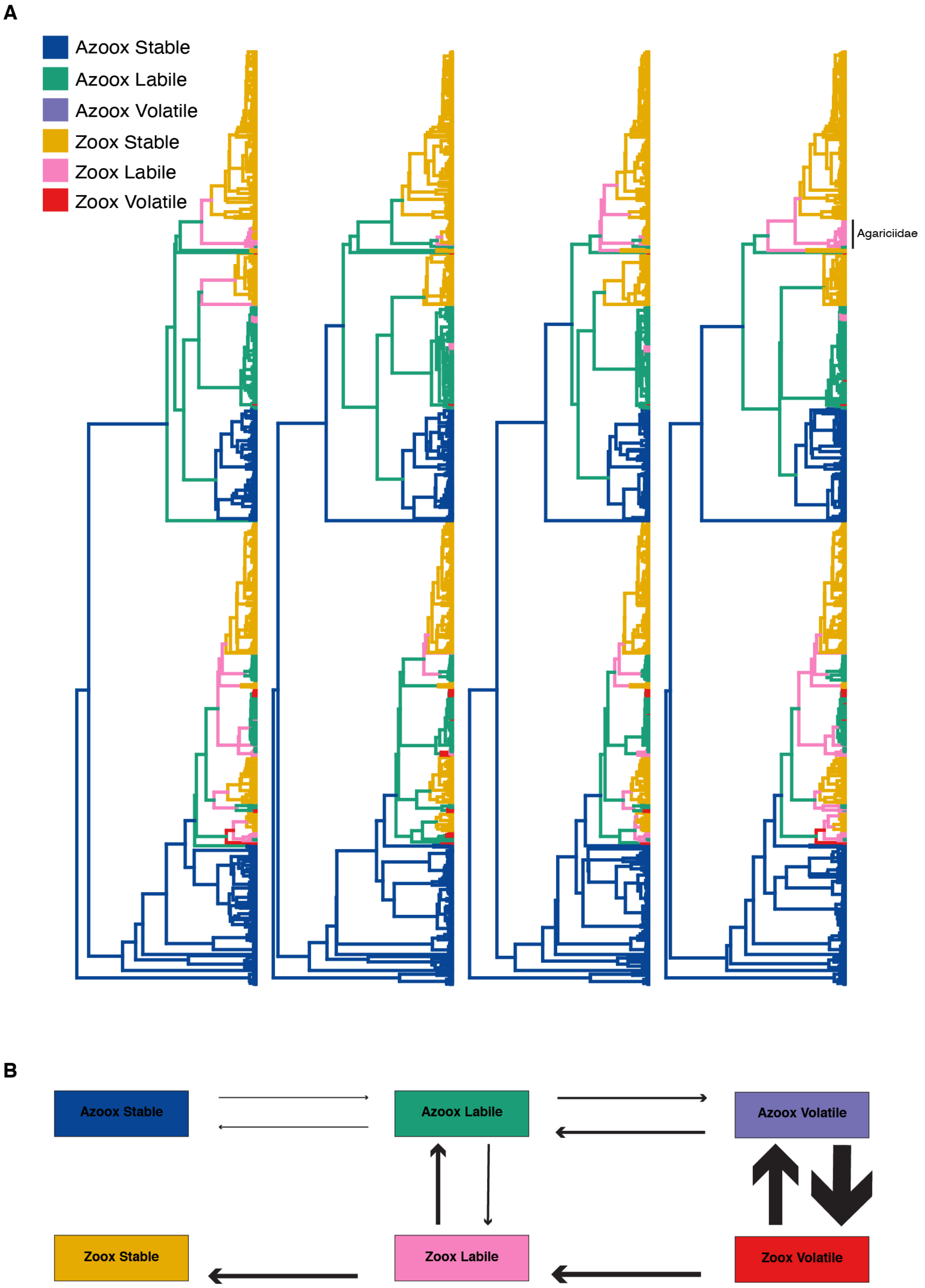

Fig. S2. Sample of the 158 supertree phylogenies where Azoox Labile is a transition state under the HRM+3. (A) Individual ancestral state reconstructions of state/rate-category on a

subsample of the 158 supertree phylogenies for which Zoox Labile is a transitional state. Branches are colored according to the most likely state at their ancestral node. **(B)** Corresponding rate diagram calculated from the sample. Rates have been omitted. Arrow width corresponds to the relative magnitudes of the rates.

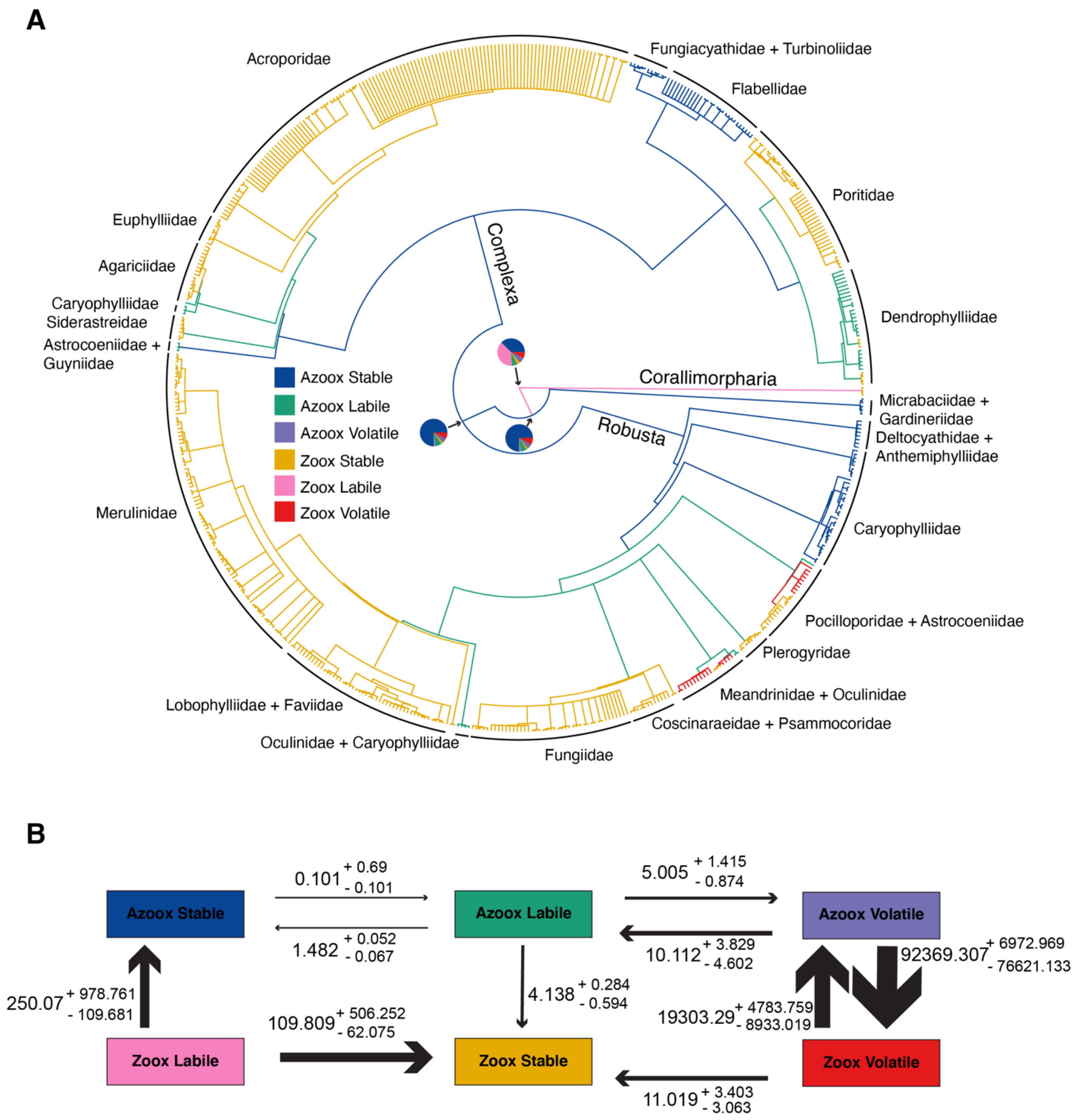

Fig. S3. Molecular tree ancestral state reconstruction and transition rates estimated under the HRM+3. (A) Ancestral state reconstruction of each state/rate-category combination inferred across 100 randomly sampled molecular phylogenies under the HRM+3. Phylogeny shown is the 95% consensus tree of the100 sampled molecular phylogenies. To calculate the probability at each internal node the mean of each state/rate-category across all 100 phylogenies was calculated for all nodes that are bifurcating in the 95% consensus tree. Pie charts at selected nodes show the probability of being in each state/rate-category. Branches are painted according to the state that is most likely at each internal node. **(B)** Schematic version of the transition matrix of the HRM+3 fit to the 100 molecular phylogenies. Transition rates printed here are the median of the transition

rates estimated for the 100 molecular phylogenies. Errors printed here represent the 95% quantile around the median as estimated via bootstrapping. Median values and errors are multiplied by 1000 to aid interpretation. The width of the arrows corresponds to relative magnitude of the rates.

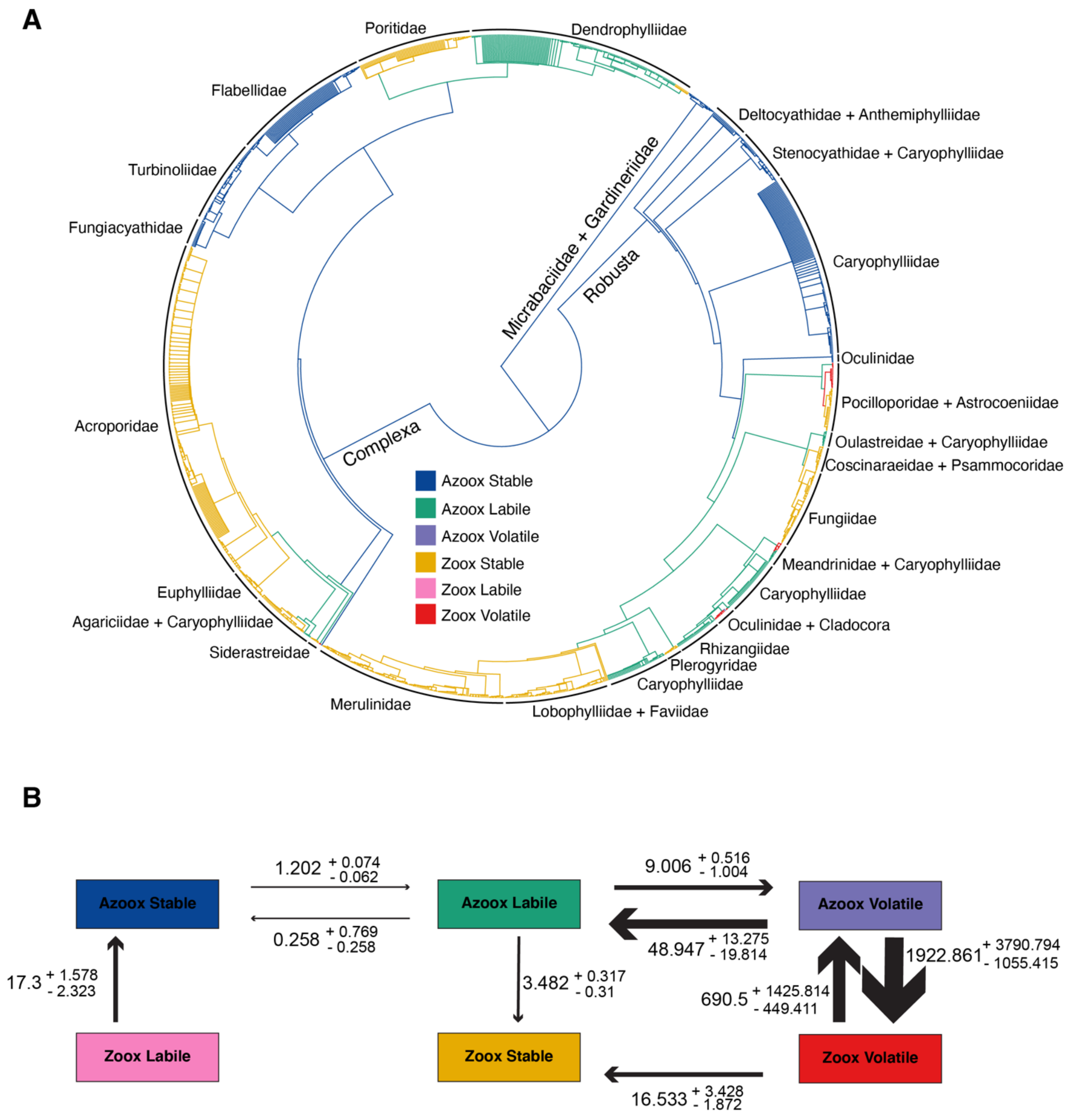

Fig. S4. Supertree ancestral state reconstruction and transition rates estimated under the HRM+3: Facultative corals pruned. (A) Ancestral state reconstruction of each state/rate-category combination inferred across 100 randomly sampled supertree phylogenies under the HRM+3 with facultative species pruned. Phylogeny shown is the 95% consensus tree of the 100 supertree phylogenies. To calculate the probability at each internal node the mean of each state/rate-category across all 100 phylogenies was calculated for all nodes that are bifurcating in the 95% consensus tree. Branches are painted according to the state that is most likely at each internal node. **(B)** Schematic version of the transition matrix of the HRM+3 fit to the 100 supertree phylogenies with facultative species pruned. Transition rates printed here are the

median of the transition rates estimated for the 100 supertree phylogenies. Errors printed here represent the 95% quantile around the median as estimated via bootstrapping. Median values and errors are multiplied by 1000 to aid interpretation. The width of the arrows corresponds to relative magnitude of the rates.

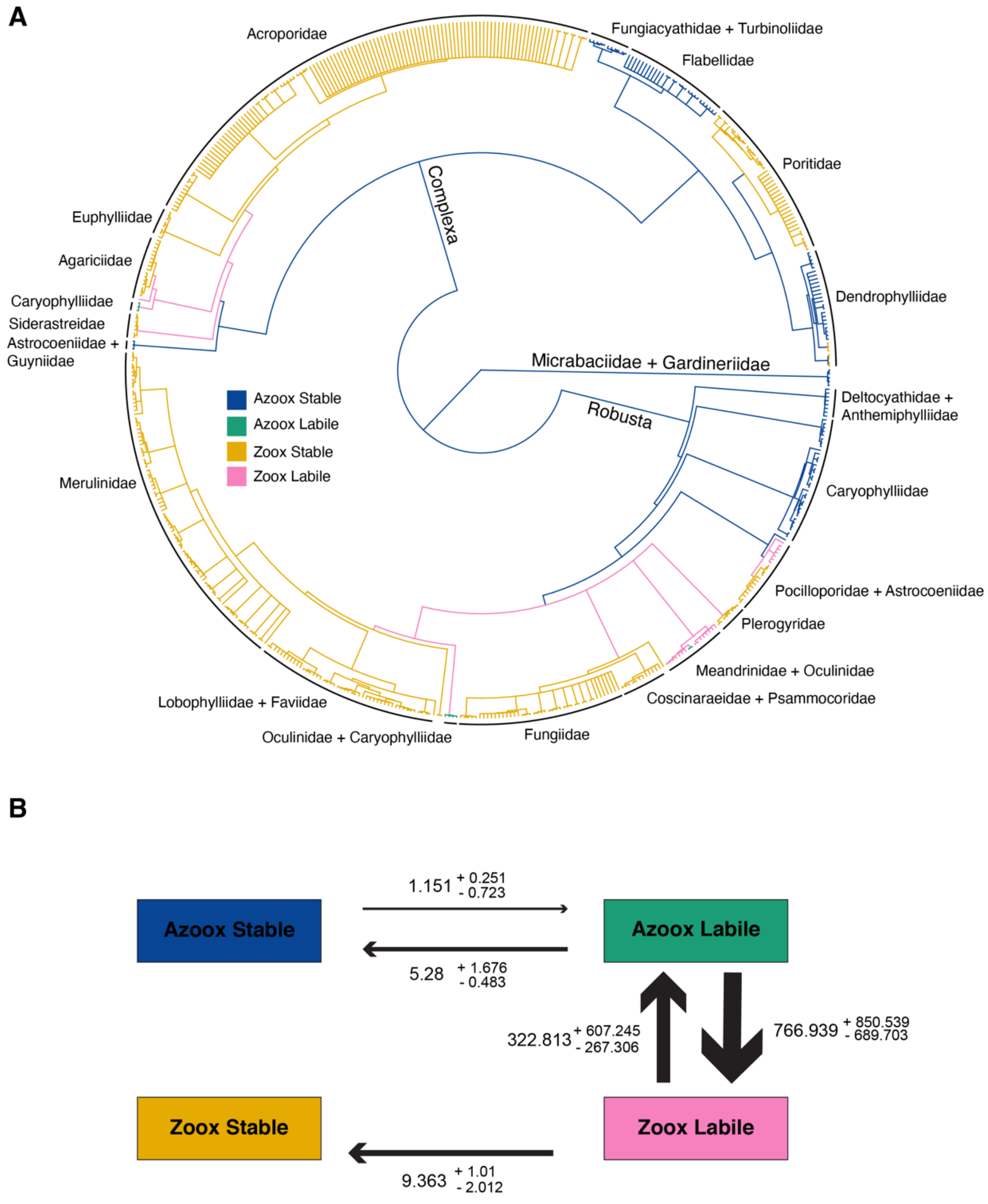

Fig. S5. Molecular tree ancestral state reconstruction and transition rates estimated under the HRM+2: Facultative corals pruned. (A) Ancestral state reconstruction of each state/rate-category combination inferred across100 randomly sampled molecular phylogenies under the HRM+2 with facultative species pruned. Phylogeny shown is the 95% consensus tree of the 100 molecular phylogenies. To calculate the probability at each internal node the mean of each state/rate-category across all 100 phylogenies was calculated for all nodes that are bifurcating in the 95% consensus tree. Branches are painted according to the state that is most likely at each internal node. **(B)** Schematic version of the transition matrix of the HRM+2 fit to the 100

molecular phylogenies with facultative species pruned. Transition rates printed here are the median of the transition rates estimated for the 100 molecular phylogenies. Errors printed here represent the 95% quantile around the median as estimated via bootstrapping. Median values and errors are multiplied by 1000 to aid interpretation. The width of the arrows corresponds to relative magnitude of the rates.

Fig. S6. (see external file abh4243_Suppl. Other File Type_seq1_v2.pdf)
Detailed ancestral state reconstruction of each state/rate-category combination inferred across the 1000 supertree phylogenies under the HRM+3. Phylogeny shown is the 95% consensus tree of all 1000 supertree phylogenies used for the analysis. To calculate the probability at each internal node the mean of each state/rate-category across all 1000 phylogenies was calculated for all nodes that are bifurcating in the 95% consensus tree. Pie charts show the probability of being in each state/rate-category. Branches are painted according to the state that is most likely at each internal node. Tips are labeled according to species.

Fig. S7. (see external file abh4243_Suppl. Other File Type_seq2_v2.pdf)
Detailed ancestral state reconstruction of each state/rate-category combination inferred across the 3,361 molecular phylogenies under the HRM+2. Phylogeny shown is the 95% consensus tree of all 3,361 molecular phylogenies used for the analysis. To calculate the probability at each internal node the mean of each state/rate-category across all 3,361 phylogenies was calculated for all nodes that are bifurcating in the 95% consensus tree. Pie charts show the probability of being in each state/rate-category. Branches are painted according to the state that is most likely at each internal node. Tips are labeled according to species.

Table S1. (See external file abh4243_Suppl. Other File Type_seq3_v2.csv) Table of species included in the supertree with corresponding observed state (AZ: Azooxanthellate, Z: Zooxanthellate, F: Facultative) and estimated probability of being in each rate category.

Table S2. (See external file abh4243_Suppl. Other File Type_seq4_v2.csv) Table of species included in the molecular tree with corresponding observed state (AZ: Azooxanthellate, Z: Zooxanthellate, F: Facultative) and estimated probability of being in each rate category.

REFERENCES AND NOTES

1. N. A. Moran, Symbiosis as an adaptive process and source of phenotypic complexity. *Proc. Natl. Acad. Sci. U.S.A.* **104**, 8627–8633 (2007).
2. A. E. Douglas, *The Symbiotic Habit* (Princeton Univ. Press, 2010).
3. J. L. Sachs, E. L. Simms, Pathways to mutualism breakdown. *Trends Ecol. Evol.* **21**, 585–592 (2006).
4. T. C. LaJeunesse, J. E. Parkinson, P. W. Gabrielson, H. J. Jeong, J. D. Reimer, C. R. Voolstra, S. R. Santos, Systematic revision of Symbiodiniaceae highlights the antiquity and diversity of coral endosymbionts. *Curr. Biol.* **28**, 2570–2580.e6 (2018).
5. J. S. Madin, K. D. Anderson, M. H. Andreasen, T. C. L. Bridge, S. D. Cairns, S. R. Connolly, E. S. Darling, M. Diaz, D. S. Falster, E. C. Franklin, R. D. Gates, A. M. T. Harmer, M. O. Hoogenboom, D. Huang, S. A. Keith, M. A. Kosnik, C.-Y. Kuo, J. M. Lough, C. E. Lovelock, O. Luiz, J. Martinelli, T. Mizerek, J. M. Pandolfi, X. Pochon, M. S. Pratchett, H. M. Putnam, T. E. Roberts, M. Stat, C. C. Wallace, E. Widman, A. H. Baird, The Coral Trait Database, a curated database of trait information for coral species from the global oceans. *Scientific Data.* **3**, 160017 (2016).
6. M. Stat, D. Carter, O. Hoegh-Guldberg, The evolutionary history of *Symbiodinium* and scleractinian hosts—Symbiosis, diversity, and the effect of climate change. *Perspect. Plant Ecol. Evol. Syst.* **8**, 23–43 (2006).
7. S. Iwasaki, M. Inoue, A. Suzuki, O. Sasaki, H. Kano, A. Iguchi, K. Sakai, H. Kawahata, The role of symbiotic algae in the formation of the coral polyp skeleton: 3-D morphological study based on x-ray microcomputed tomography. *Geochem. Geophys. Geosyst.* **17**, 3629–3637 (2016).
8. L. Plaisance, M. J. Caley, R. E. Brainard, N. Knowlton, The diversity of coral reefs: What are we missing? *PLOS ONE* **6**, e25026 (2011).
9. S. D. Cairns, Phylogenetic list of 722 valid Recent azooxanthellate scleractinian species, with their junior synonyms and depth ranges. Supplement to *Cold-Water Corals: The Biology and Geology of Deep-Sea Coral Habitats*, J. M. Roberts, A. Wheeler, A. Freiwald, S. Cairns, Eds. (Cambridge Univ. Press, 2009); https://lophelia.org/images/stories/pdfs/Cold-water_Corals_Online_Appendix.pdf.
10. K. P. Leydet, M. E. Hellberg, Discordant coral–symbiont structuring: Factors shaping geographical variation of *Symbiodinium* communities in a facultative zooxanthellate coral genus, Oculina. *Coral Reefs.* **35**, 583–595 (2016).
11. J. Dimond, E. Carrington, Temporal variation in the symbiosis and growth of the temperate scleractinian coral *Astrangia poculata*. *Mar. Ecol. Prog. Ser.* **348**, 161–172 (2007).

12. M. S. Barbeitos, S. L. Romano, H. R. Lasker, Repeated loss of coloniality and symbiosis in scleractinian corals. *Proc. Natl. Acad. Sci. U.S.A.* **107**, 11877–11882 (2010).
13. A. N. Campoy, A. M. Addamo, A. Machordom, A. Meade, M. M. Rivadeneira, C. E. Hernández, C. Venditti, The origin and correlated evolution of symbiosis and coloniality in scleractinian corals. *Front. Mar. Sci.* **7**, 461 (2020).
14. D. Huang, K. Roy, The future of evolutionary diversity in reef corals. *Philos. Trans. R. Soc. Lond., B, Biol. Sci.* **370**, 20140010 (2015).
15. M. V. Kitahara, H. Fukami, F. Benzoni, D. Huang, The new systematics of Scleractinia: Integrating molecular and morphological evidence, in *The Cnidaria, Past, Present, and Future*, S. Goffredo, Z. Dubinsky, Eds. (Springer, 2016), pp. 41–59.
16. J. M. Beaulieu, B. C. O'Meara, M. J. Donoghue, Identifying hidden rate changes in the evolution of a binary morphological character: The evolution of plant habit in campanulid angiosperms. *Syst. Biol.* **62**, 725–737 (2013).
17. C. S. McFadden, A. M. Quattrini, M. R. Brugler, P. F. Cowman, L. F. Dueñas, M. V. Kitahara, D. A. Paz-García, J. D. Reimer, E. Rodríguez, Phylogenomics, origin, and diversification of Anthozoans (Phylum Cnidaria). *Syst. Biol.* **70**, 635–647 (2021).
18. E. Kayal, B. Bentlage, M. Sabrina Pankey, A. H. Ohdera, M. Medina, D. C. Plachetzki, A. G. Collins, J. F. Ryan, Phylogenomics provides a robust topology of the major cnidarian lineages and insights on the origins of key organismal traits. *BMC Evol. Biol.* **18**, 68 (2018).
19. J. Stolarski, M. V. Kitahara, D. J. Miller, S. D. Cairns, M. Mazur, A. Meibom, The ancient evolutionary origins of Scleractinia revealed by azooxanthellate corals. *BMC Evol. Biol.* **11**, 316 (2011).
20. M. V. Kitahara, S. D. Cairns, J. Stolarski, D. Blair, D. J. Miller, A comprehensive phylogenetic analysis of the Scleractinia (Cnidaria, Anthozoa) based on mitochondrial CO1 sequence data. *PLOS ONE* **5**, e11490 (2010).
21. Y. Ezaki, The Permian coral *Numidiaphyllum*: New insights into anthozoan phylogeny and Triassic sceleractinian origins. *Palaeontology.* **40**, 1–14 (1997).
22. Y. Ezaki, Paleozoic Scleractinia: Progenitors or extinct experiments? *Paleobiology* **24**, 227–234 (1998).
23. Y. Ezaki, Palaeoecological and phylogenetic implications of a new scleractiniamorph genus from Permian sponge reefs, south China. *Palaeontology* **43**, 199–217 (2000).

24. C. T. Scrutton, E. N. Clarkson, A new scleractinian-like coral from the Ordovician of the Southern Uplands, *Scotland. Palaeontology.* **34**, 179–194 (1991).
25. J. E. N. Veron, *Corals in Space and Time: The Biogeography and Evolution of the Scleractinia* (Cornell Univ. Press, 1995).
26. E. C. E. Kvennefors, W. Leggat, O. Hoegh-Guldberg, B. M. Degnan, A. C. Barnes, An ancient and variable mannose-binding lectin from the coral *Acropora millepora* binds both pathogens and symbionts. *Dev. Comp. Immunol.* **32**, 1582–1592 (2008).
27. R. Cunning, R. A. Bay, P. Gillette, A. C. Baker, N. Traylor-Knowles, Comparative analysis of the *Pocillopora damicornis* genome highlights role of immune system in coral evolution. *Sci. Rep.* **8**, 16134 (2018).
28. M. Hamada, E. Shoguchi, C. Shinzato, T. Kawashima, D. J. Miller, N. Satoh, The complex NOD-like receptor repertoire of the coral *Acropora digitifera* includes novel domain combinations. *Mol. Biol. Evol.* **30**, 167–176 (2013).
29. C. Shinzato, E. Shoguchi, T. Kawashima, M. Hamada, K. Hisata, M. Tanaka, M. Fujie, M. Fujiwara, R. Koyanagi, T. Ikuta, A. Fujiyama, D. J. Miller, N. Satoh, Using the *Acropora digitifera* genome to understand coral responses to environmental change. *Nature* **476**, 320–323 (2011).
30. E. Meyer, V. M. Weis, Study of cnidarian-algal symbiosis in the "omics" age. *Biol. Bull.* **223**, 44–65 (2012).
31. S. K. Davy, D. Allemand, V. M. Weis, Cell biology of cnidarian-dinoflagellate symbiosis. *Microbiol. Mol. Biol. Rev.* **76**, 229–261 (2012).
32. R. C. Baron-Szabo, Corals of the K/T-boundary: Scleractinian corals of the suborders Dendrophylliina, Caryophylliina, Fungiina, Microsolenina, and Stylinina. *Zootaxa* **1952**, 1–244 (2008).
33. R. C. Baron-Szabo, Corals of the K/T-boundary: Scleractinian corals of the suborders Astrocoeniina, Faviina, Rhipidogyrina and Amphiastraeina. *J. Syst. Palaeontol.* **4**, 1–108 (2006).
34. E. A. Herre, N. Knowlton, U. G. Mueller, S. A. Rehner, The evolution of mutualisms: Exploring the paths between conflict and cooperation. *Trends Ecol. Evol.* **14**, 49–53 (1999).
35. A. C. Hartmann, A. H. Baird, N. Knowlton, D. Huang, The paradox of environmental symbiont acquisition in obligate mutualisms. *Curr. Biol.* **27**, 3711–3716.e3 (2017).

36. R. N. Silverstein, A. M. S. Correa, A. C. Baker, Specificity is rarely absolute in coral–algal symbiosis: Implications for coral response to climate change. *Proc. R. Soc. B* **279**, 2609–2618 (2012).
37. N. S. Fabina, H. M. Putnam, E. C. Franklin, M. Stat, R. D. Gates, Transmission mode predicts specificity and interaction patterns in coral-*Symbiodinium* networks. *PLOS ONE* **7**, e44970 (2012).
38. T. D. Swain, M. W. Westneat, V. Backman, L. A. Marcelino, Phylogenetic analysis of symbiont transmission mechanisms reveal evolutionary patterns in thermotolerance and host specificity that enhance bleaching resistance among vertically transmitted *Symbiodinium*. *Eur. J. Phycol.* **53**, 443–459 (2018).
39. H. Ying, I. Cooke, S. Sprungala, W. Wang, D. C. Hayward, Y. Tang, G. Huttley, E. E. Ball, S. Forêt, D. J. Miller, Comparative genomics reveals the distinct evolutionary trajectories of the robust and complex coral lineages. *Genome Biol.* **19**, 175 (2018).
40. C. Shinzato, S. Mungpakdee, N. Satoh, E. Shoguchi, A genomic approach to coral-dinoflagellate symbiosis: Studies of *Acropora digitifera* and *Symbiodinium minutum*. *Front. Microbiol.* **5**, 336 (2014).
41. S. J. Robbins, C. M. Singleton, C. X. Chan, L. F. Messer, A. U. Geers, H. Ying, A. Baker, S. C. Bell, K. M. Morrow, M. A. Ragan, D. J. Miller, S. Forêt, C. R. Voolstra, G. W. Tyson, D. G. Bourne, D. G. Bourne, A genomic view of the reef-building coral *Porites lutea* and its microbial symbionts. *Nat. Microbiol.* **4**, 2090–2100 (2019).
42. G. D. Stanley Jr.. P. K. Swart, Evolution of the coral-zooxanthellae symbiosis during the Triassic: A geochemical approach. *Paleobiology* **21**, 179–199 (1995).
43. K. Frankowiak, X. T. Wang, D. M. Sigman, A. M. Gothmann, M. V. Kitahara, M. Mazur, A. Meibom, J. Stolarski, Photosymbiosis and the expansion of shallow-water corals. *Sci. Adv.* **2**, e1601122 (2016).
44. C. Simpson, W. Kiessling, H. Mewis, R. C. Baron-Szabo, J. Müller, Evolutionary diversification of reef corals: A comparison of the molecular and fossil records. *Evolution* **65**, 3274–3284 (2011).
45. G. D. Stanley Jr., Photosymbiosis and the evolution of modern coral reefs. *Science* **312**, 857–858 (2006).
46. A. F. Budd, S. L. Romano, N. D. Smith, M. S. Barbeitos, Rethinking the phylogeny of scleractinian corals: A review of morphological and molecular data. *Integr. Comp. Biol.* **50**, 411–427 (2010).

47. J. Stolarski, E. Roniewicz, Towards a new synthesis of evolutionary relationships and classification of Scleractinia. *J. Paleo.* **75**, 1090–1108 (2001).
48. A. M. Quattrini, E. Rodríguez, B. C. Faircloth, P. F. Cowman, M. R. Brugler, G. A. Farfan, M. E. Hellberg, M. V. Kitahara, C. L. Morrison, D. A. Paz-García, J. D. Reimer, C. S. McFadden, Palaeoclimate ocean conditions shaped the evolution of corals and their skeletons through deep time. *Nat. Ecol. Evol.* **4**, 1531–1538 (2020).
49. D. Huang, Threatened reef corals of the world. *PLOS ONE* **7**, e34459 (2012).
50. D. Huang, K. Roy, Anthropogenic extinction threats and future loss of evolutionary history in reef corals. *Ecol. Evol.* **3**, 1184–1193 (2013).
51. R. Bouckaert, J. Heled, D. Kühnert, T. Vaughan, C.-H. Wu, D. Xie, M. A. Suchard, A. Rambaut, A. J. Drummond, BEAST 2: A software platform for bayesian evolutionary analysis. *PLOS Comput. Biol.* **10**, e1003537 (2014).
52. R Core Team, *R: A Language and Environment for Statistical Computing* (R Foundation for Statistical Computing, 2018); www.R-project.org/.
53. M. Plummer, N. Best, K. Cowles, K. Vines, CODA: Convergence Diagnosis and Output Analysis for MCMC. *R News.* **6**, 7–11 (2006).
54. T. Horton, A. Kroh, S. Ahyong, N. Bailly, N. Boury-Esnault, S. N. Brandão, M. J. Costello, S. Gofas, F. Hernandez, J. Mees, G. Paulay, G. C. B. Poore, G. Rosenberg, W. Decock, S. Dekeyzer, T. Lanssens, L. Vandepitte, B. Vanhoorne, K. Verfaille, R. Adlard, P. Adriaens, S. Agatha, K. J. Ahn, N. Akkari, B. Alvarez, G. Anderson, M. Angel, C. Arango, T. Artois, S. Atkinson, R. Bank, A. Barber, J. P. Barbosa, I. Bartsch, D. Bellan-Santini, J. Bernot, A. Berta, R. Bieler, S. Blanco, I. Blasco-Costa, M. Blazewicz, P. Bock, R. Böttger-Schnack, P. Bouchet, G. Boxshall, C. B. Boyko, R. Bray, B. Breure, N. L. Bruce, S. Cairns, T. N. Campinas Bezerra, P. Cárdenas, E. Carstens, B. K. Chan, T. Y. Chan, L. Cheng, M. Churchill, C. O. Coleman, A. G. Collins, L. Corbari, R. Cordeiro, A. Cornils, M. Coste, K. A. Crandall, T. Cribb, S. Cutmore, F. Dahdouh-Guebas, M. Daly, M. Daneliya, J. C. Dauvin, P. Davie, C. De Broyer, S. De Grave, V. de Mazancourt, N. de Voogd, P. Decker, W. Decraemer, D. Defaye, J. L. d'Hondt, H. Dijkstra, M. Dohrmann, J. Dolan, D. Domning, R. Downey, I. Drapun, L. Ector, U. Eisendle-Flöckner, M. Eitel, S. C. d. Encarnação, H. Enghoff, J. Epler, C. Ewers-Saucedo, M. Faber, S. Feist, D. Figueroa, J. Finn, C. Fišer, E. Fordyce, W. Foster, J. H. Frank, C. Fransen, H. Furuya, H. Galea, O. Garcia-Alvarez, R. Garic, R. Gasca, S. Gaviria-Melo, S. Gerken, H. Gheerardyn, D. Gibson, J. Gil, A. Gittenberger, C. Glasby, A. Glover, S. E. Gómez-

Noguera, D. González-Solís, D. Gordon, M. Grabowski, C. Gravili, J. M. Guerra-García, R. Guidetti, M. D. Guiry, K. A. Hadfield, E. Hajdu, J. Hallermann, B. Hayward, E. Hendrycks, D. Herbert, A. Herrera Bachiller, J. s. Ho, J. Høeg, B. Hoeksema, O. Holovachov, J. Hooper, R. Houart, L. Hughes, M. Hyžný, L. F. M. Iniesta, T. Iseto, S. Ivanenko, M. Iwataki, G. Jarms, D. Jaume, K. Jazdzewski, P. Jóźwiak, Y. Kantor, I. Karanovic, B. Karthick, Y. H. Kim, R. King, P. M. Kirk, M. Klautau, J. P. Kociolek, F. Köhler, J. Kolb, A. Kotov, T. Krapp-Schickel, A. Kremenetskaia, R. Kristensen, M. Kulikovskiy, S. Kullander, R. La Perna, G. Lambert, D. Lazarus, F. Le Coze, S. LeCroy, D. Leduc, E. J. Lefkowitz, R. Lemaitre, Y. Liu, A. N. Lörz, J. Lowry, T. Ludwig, N. Lundholm, E. Macpherson, L. Madin, C. Mah, T. Mamos, R. Manconi, G. Mapstone, P. E. Marek, B. Marshall, D. J. Marshall, P. Martin, S. McInnes, T. Meidla, K. Meland, K. Merrin, R. Mesibov, C. Messing, D. Miljutin, C. Mills, Ø. Moestrup, V. Mokievsky, T. Molodtsova, F. Monniot, R. Mooi, A. C. Morandini, R. Moreira da Rocha, F. Moretzsohn, J. Mortelmans, J. Mortimer, L. Musco, T. A. Neubauer, E. Neubert, B. Neuhaus, P. Ng, A. D. Nguyen, C. Nielsen, T. Nishikawa, J. Norenburg, T. O'Hara, D. Opresko, M. Osawa, Y. Ota, B. Páll-Gergely, D. Patterson, H. Paxton, R. Peña Santiago, V. Perrier, W. Perrin, I. Petrescu, B. Picton, J. F. Pilger, A. Pisera, D. Polhemus, M. Potapova, P. Pugh, G. Read, M. Reich, J. D. Reimer, H. Reip, M. Reuscher, J. W. Reynolds, I. Richling, F. Rimet, P. Ríos, M. Rius, D. C. Rogers, K. Rützler, A. Rzhavsky, K. Sabbe, J. Saiz-Salinas, S. Sala, S. Santos, E. Sar, A. F. Sartori, A. Satoh, H. Schatz, B. Schierwater, A. Schmidt-Rhaesa, S. Schneider, C. Schönberg, P. Schuchert, A. R. Senna, C. Serejo, S. Shaik, S. Shamsi, J. Sharma, W. A. Shear, N. Shenkar, A. Shinn, M. Short, J. Sicinski, V. Siegel, P. Sierwald, E. Simmons, F. Sinniger, D. Sivell, B. Sket, H. Smit, N. Smit, N. Smol, J. F. Souza-Filho, J. Spelda, W. Sterrer, E. Stienen, P. Stoev, S. Stöhr, M. Strand, E. Suárez-Morales, M. Summers, C. Suttle, B. J. Swalla, S. Taiti, M. Tanaka, A. H. Tandberg, D. Tang, M. Tasker, J. Taylor, J. Taylor, A. Tchesunov, H. ten Hove, J. J. ter Poorten, J. Thomas, E. V. Thuesen, M. Thurston, B. Thuy, J. T. Timi, T. Timm, A. Todaro, X. Turon, S. Tyler, P. Uetz, S. Utevsky, J. Vacelet, D. Vachard, W. Vader, R. Väinölä, B. Van de Vijver, S. E. van der Meij, T. van Haaren, R. van Soest, R. Van Syoc, A. Vanreusel, V. Venekey, M. Vinarski, R. Vonk, C. Vos, G. Walker-Smith, T. C. Walter, L. Watling, M. Wayland, T. Wesener, C. Wetzel, C. Whipps, K. White, D. Williams, G. Williams, R. Wilson, A. Witkowski, J. Witkowski, N. Wyatt, C. Wylezich, K. Xu, J. Zanol, W. Zeidler, Z. Zhao, World Register of Marine Species (WoRMS) (2018); www.marinespecies.org.

55. S. Chamberlain, *worrms: World Register of Marine Species (WoRMS) Client* (2018); https://CRAN.R-project.org/package=worrms.
56. J. M. Beaulieu, J. C. Oliver, B. C. O'Meara, *corHMM: Analysis of Binary Character Evolution* (2017); https://CRAN.R-project.org/package=corHMM.
57. R. Pordes, D. Petravick, B. Kramer, D. Olson, M. Livny, A. Roy, P. Avery, K. Blackburn, T. Wenaus, F. Würthwein, in *Journal of Physics: Conference Series* (IOP Publishing, 2007), vol. 78, p. 012057.
58. I. Sfiligoi, D. C. Bradley, B. Holzman, P. Mhashilkar, S. Padhi, F. Wurthwein, in *2009 WRI World Congress on Computer Science and Information Engineering* (IEEE, 2009), vol. 2, pp. 428–432.
59. B. W. Hoeksema, Forever in the dark: the cave-dwelling azooxanthellate reef coral *Leptoseris troglodyta* sp. n.(Scleractinia, Agariciidae). *Zookeys* **228**, 21–37 (2012).
