# Supplementary figures and images for: Lineage-specific variation in the evolutionary stability of coral photosymbiosis
Source: Sci Adv. 2021 Sep 22;7(39):eabh4243. doi: 10.1126/sciadv.abh4243 (PMC8457658; doi:10.1126/sciadv.abh4243)

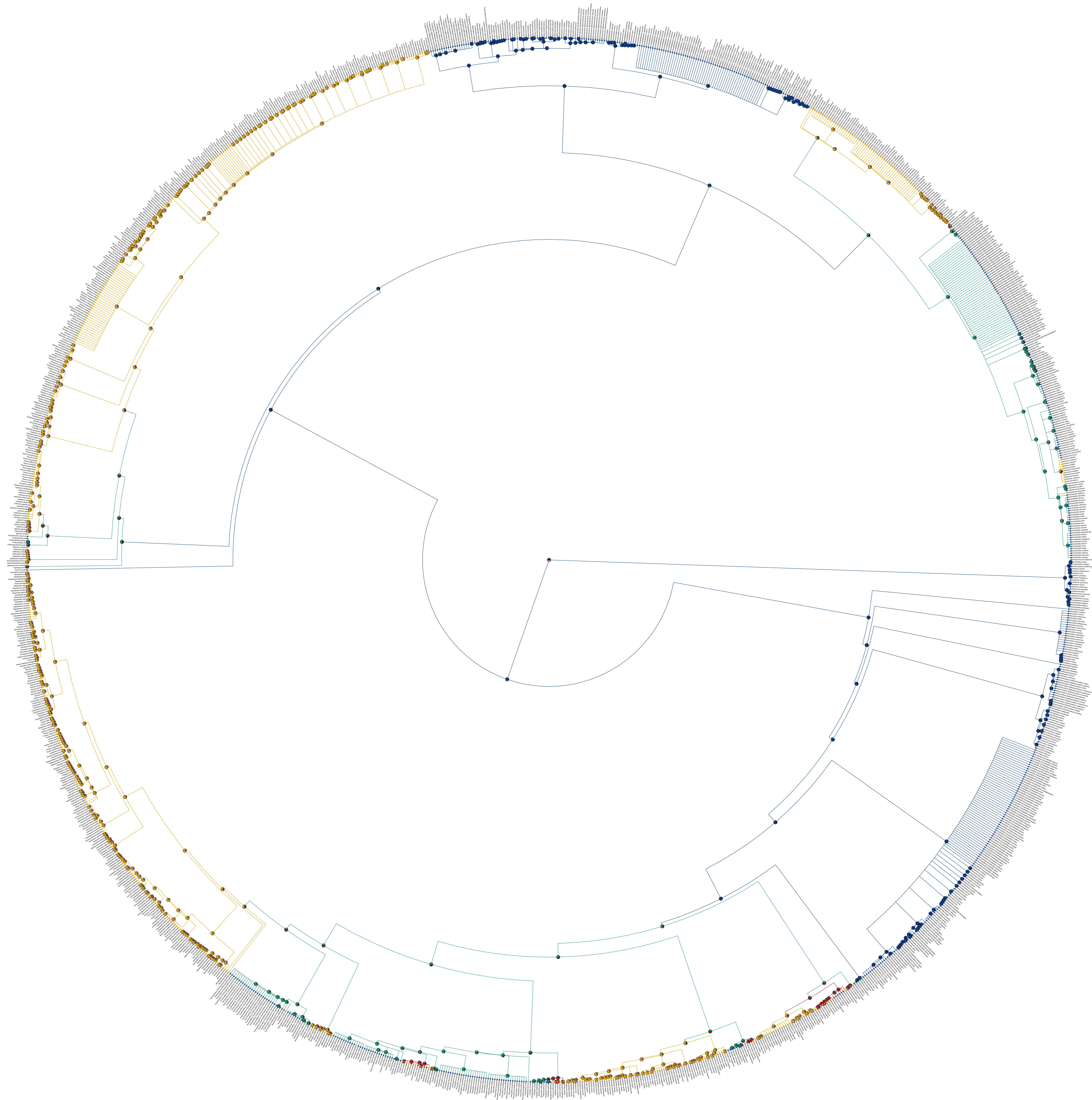

Supplement: Supplementary file 2 — Figs. S6 and S7 [file sciadv.abh4243_figures_s6_and_s7.zip › sciadv.abh4243_Figure_S6.pdf]

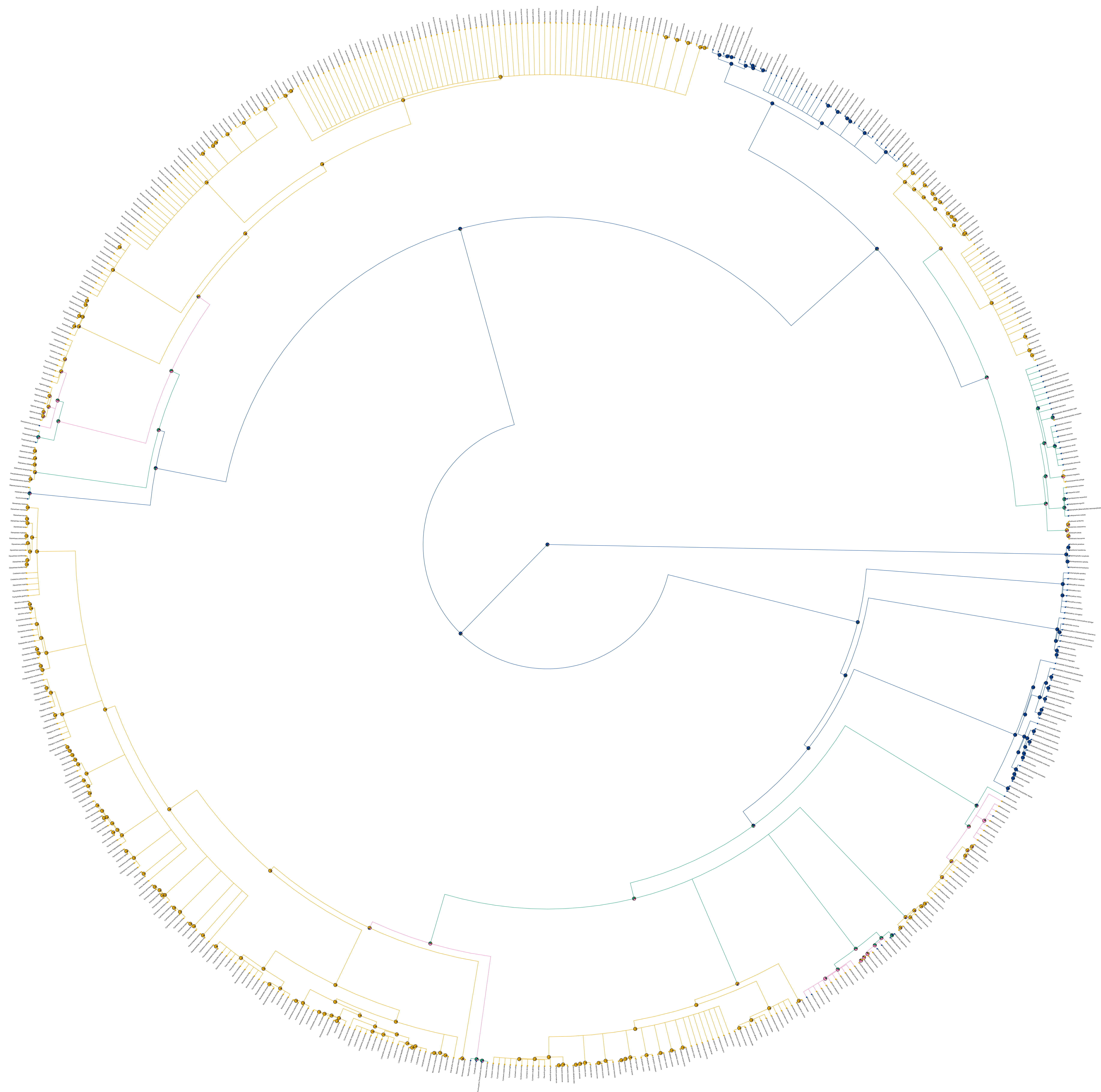

Supplement: Supplementary file 2 — Figs. S6 and S7 [file sciadv.abh4243_figures_s6_and_s7.zip › sciadv.abh4243_Figure_S7.pdf]
